# Decreased FANCJ caused by 5FU contributes to the increased sensitivity to oxaliplatin in gastric cancer cells

**DOI:** 10.1007/s10120-012-0191-0

**Published:** 2012-09-12

**Authors:** Ryutaro Mori, Kazuhiro Yoshida, Toshiyuki Tanahashi, Kazunori Yawata, Junko Kato, Naoki Okumura, Yasuhiro Tsutani, Morihito Okada, Naohide Oue, Wataru Yasui

**Affiliations:** 1 Department of Surgical Oncology, Gifu University, Graduate School of Medicine, 1-1 Yanagido, Gifu, Gifu 501-1194 Japan; 2 Department of Surgical Oncology, Research Institute for Radiation Biology and Medicine, Hiroshima University, Hiroshima, Japan; 3 Department of Molecular Pathology, Hiroshima University Graduate School of Medicine, Hiroshima, Japan

**Keywords:** Fluorouracil, Oxaliplatin, BACH1 protein

## Abstract

**Background:**

Oxaliplatin is effective against many types of cancer, and the combination of 5-fluorouracil (5FU) and oxaliplatin is synergistically effective against gastric cancer, as well as colon cancer. The FANCJ protein is one of the Fanconi anemia (FA) gene products, and its interaction with the tumor suppressor BRCA1 is required for DNA double-strand break (DSB) repair. FANCJ also functions in interstrand crosslinks (ICLs) repair by linking to mismatch repair protein complex MLH1-PMS2 (MutLα). While oxaliplatin causes ICLs, 5FU is considered to cause DSBs. Therefore, we investigated the importance of FANCJ in the synergistic effects of oxaliplatin and 5FU in MKN45 gastric cancer cells and the derived 5FU-resistant cell line, MKN45/F2R.

**Methods:**

MKN1, TMK1, MKN45, and MKN45/F2R (5FU-resistant) gastric cancer cells were treated with 5FU and/or oxaliplatin. The signaling pathway was evaluated by a western blotting analysis and reverse transcription polymerase chain reaction (RT-PCR). Drug resistance was evaluated by the 3-(4,5-dimethyl-2-tetrazolyl)-2,5-diphenyl-2H tetrazolium bromide (MTT) assay.

**Results:**

In MKN45 cells, the combination of 5FU and oxaliplatin had synergistic effects. DSBs appeared when the cells were treated with 5FU. FANCJ was down-regulated, and BRCA1 was induced in a dose- and time-dependent manner. MKN45 cells showed increased sensitivity to oxaliplatin when FANCJ was knocked down by short interfering (si) RNA. However, these findings were not observed in MKN45/F2R 5FU-resistant cells.

**Conclusion:**

These results strongly suggest that the decrease in FANCJ caused by 5FU treatment leads to an increase in sensitivity to oxaliplatin, thus indicating that the FANCJ protein plays an important role in the synergism of the combination of 5FU and oxaliplatin.

## Introduction

Gastric cancer remains one of the major causes of cancer deaths around the world [1, 2]. Most patients with advanced and metastatic gastric cancer are treated with chemotherapy, and the combination of S-1 and cisplatin (CDDP) is one of the standard first-line regimens used in Japan [3].

The combination of fluorouracil (5FU) and oxaliplatin is used in the fluorouracil, leucovorin, and oxaliplatin (FOLFOX) regimen for colorectal cancer, and its efficacy has been clinically confirmed [4]. Oxaliplatin exerts growth inhibitory effects on many cancer cell lines and tumors, including some that are primarily resistant to CDDP and carboplatin. This increased activity is due to its 1, 2-diaminocyclohexane (DACH) carrier ligand, which provides higher lipophilicity, as evidenced by its large volume of distribution and slow excretion through the kidneys [5]. The combination of 5FU and oxaliplatin against gastric cancer has been demonstrated to be effective in the clinic [6, 7], and oxaliplatin is sometimes used to replace CDDP for the treatment of gastric cancer, because of its better tolerability [8]. Oxaliplatin and 5FU have demonstrated activity against colon cancer cell lines, and synergistic activity between the agents has been observed in experimental models [9, 10], but the mechanism underlying their synergistic effect is unclear.

The FANCJ protein is one of the Fanconi anemia (FA) gene products. It was first identified as a protein that binds directly to the breast cancer-associated tumor suppressor, BRCA1 [11, 12], and was originally named BACH1/BRIP1 [12, 13]. Fanconi anemia is a rare hereditary disorder characterized by skeletal abnormalities, bone marrow failure, and an increased incidence of cancer. The basic cellular abnormality in FA has been postulated to lie in the DNA repair mechanisms, because cells from FA patients display chromosomal abnormalities and are hypersensitive to agents that cause DNA interstrand crosslinks (ICLs), such as mitomycin C (MMC) and CDDP [14]. The role of FANCJ in the FA pathway has not yet been completely elucidated. So far, it has been shown that FANCJ is a DNA helicase for the D-loop structure in the early stage of the homologous recombination (HR) pathway of double-strand break (DSB) repair; therefore, the association of FANCJ with BRCA1 is essential for DSB repair [12, 13]. Moreover, FANCJ interacts with the mismatch repair complex MutLα, composed of MLH1 and PMS2, independent of BRCA1, and the FANCJ/MutLα interaction is essential for ICL repair [15].

It is known that 5FU induces DSBs as a result of its incorporation into DNA [16] or thimidylate synthase (TS) inhibition [17], and oxaliplatin induces ICLs by its pharmacological action. Based on these facts, we hypothesized that the two functions of FANCJ would be involved in the synergistic effects of 5FU and oxaliplatin against gastric cancer.

In the present study, we clarified the differential regulation of the FANCJ protein between 5FU-sensitive and 5FU-resistant cells and also demonstrated the mechanism underlying the synergistic effects of 5FU and oxaliplatin against gastric cancer cells.

## Materials and methods

### Drugs

5FU was purchased from Kyowa Hakko (Tokyo, Japan), and oxaliplatin was purchased from Yakult Honsha (Tokyo, Japan).

### Cell lines and cell culture

Gastric cancer cell lines (MKN45, MKN1, TMK1) were cultured in RPMI 1640 medium (Wako, Osaka, Japan) supplemented with 10 % fetal bovine serum (Sigma-Aldrich, St. Louis, MO, USA), antibiotics (Sigma-Aldrich), and HEPES (Sigma-Aldrich) in a humidified atmosphere of 5 % CO_2_ at 37 °C. MKN45 and TMK1 are poorly differentiated human gastric adenocarcinoma cell lines. MKN1 is an adenosquamous carcinoma cell line. MKN45/F2R is a 5FU-resistant cell line. To establish this cell line, the MKN45 parent cells were continuously exposed to increasing concentrations (0.1–2 μM) of 5FU over a period of 1 year. The MKN45/F2R cells were routinely maintained in culture medium containing 2 μM of 5FU. To eliminate the effects of 5FU in our experiments, the resistant cells were cultured in a drug-free medium for at least 2 weeks before all of the studies [18].

### 3-(4,5-Dimethyl-2-tetrazolyl)-2,5-diphenyl-2H tetrazolium bromide (MTT) assay for the effects of 5FU or oxaliplatin on cell viability

Cell growth was assessed with a standard MTT assay, which detects the dehydrogenase activity in viable cells. A total of 5 × 10^3^ cells were seeded in each well of 96-well culture plates. After 24 h, the cells were treated with various concentrations of drugs. After another 72 h, the culture medium was removed, and 100 μl of a 0.5 mg/ml solution of MTT (Sigma-Aldrich) was added to each well. The plates were then incubated for 4 h at 37 °C. The MTT solution was then removed and replaced with 100 μl of dimethyl sulfoxide (Wako) per well, and the absorbance at 540 nm was measured using an Envision 2104 Multilabel Reader (Perkin Elmer, Waltham, MA, USA).

The Combination Index (CI) was calculated by the formula CI = *A*/Ax + *B*/Bx (*A*: the 50% inhibitory concentration [IC50] for drug A in combination, *Ax*: the IC50 for drug A alone, *B*: the IC50 for drug B in combination, Bx: the IC50 for drug B alone) (based on the Loewe additivity model [19]).

### Immunofluorescence for γH2AX

The cells were harvested in a Lab-Tek Chamber Slide System (Thermo Fisher Scientific, Waltham, MA, USA) and immunofluorescence studies were performed. The cells were first fixed in 4 % paraformaldehyde for 15 min at room temperature and washed three times with phosphate-buffered saline (PBS) containing 1 % Triton X-100 (PBST). Blocking against non-specific binding was performed for 60 min with 0.5 % goat serum dissolved in PBST, and the cells were again washed three times with PBST. The rabbit monoclonal anti-phospho-H2AX antibody (Cell Signaling Technology, Danvers, MA, USA, 1:200) was used as the primary antibody. The cells were incubated for 1 h at room temperature with the primary antibody dissolved in PBST supplemented with 0.5 % goat serum, and then the cells were washed three more times with PBST. The cells were then incubated with highly cross-adsorbed Alexa Fluor 546 goat anti-rabbit IgG (Invitrogen, Carlsbad, CA, USA, 4 μg/ml), Phalloidin Alexa Fluor 488 Conjugate (Lonza, Walkersville, MD, USA, 1:40), and 4', 6-diamidino-2-phenylindole (DAPI) Nucleic Acid Stain (Invitrogen 1:25000) in PBST containing 0.5 % goat serum. Images were acquired on a DP70-WPC02 camera mounted on an IX50 system (Olympus, Tokyo, Japan).

### Immunoprecipitation, western blot analysis, and antibodies

Cells were harvested and lysed in CelLytic™ M (Sigma-Aldrich) for 30 min on ice. The protein concentration of the lysates was measured using a DC Protein Assay Kit (Bio-Rad, Hercules, CA, USA). For the immunoprecipitation assays, cell lysates were incubated with an anti-FANCJ antibody (Abcam, Cambridge, UK, 1:100) for 2 h at 4 °C and PureProteome™ Protein A Magnetic Beads (Millipore, Billerica, MA, USA) were added, and the beads were subsequently washed. The cell lysates were boiled in Sample Buffer Solution (Wako), then total cell protein extracts (20 μg/lane) were separated by sodium dodecyl sulfate–polyacrylamide gel electrophoresis using SuperSep™ (Wako), and they were electrophoretically transferred onto polyvinyl difluoride (PVDF) membranes. The membranes were blocked with PVDF blocking reagent (TOYOBO, Osaka, Japan) for 1 h. The membranes were then incubated with primary antibodies against β-actin, FANCJ, BRCA1, FANCD1/BRCA2, phospho-Histone H2AX(Ser139) (Cell Signaling Technology, 1:5000), MLH1 (Abcam, 1:100000), FANCD2 (Abcam, 1:50000), and PMS2 (EPITOMICS, San Francisco, CA, USA, 1:20000) overnight at 4 °C. The primary antibodies were diluted with Can Get Signal Solution 1 (TOYOBO). The membranes were then washed with Dako Washing Buffer (Dako, Glostrup, Denmark) and incubated with the appropriate secondary antibodies (Millipore, 1:25000). Secondary antibodies were diluted with Can Get Signal Solution 2 (TOYOBO). The immunoreactive proteins were visualized by chemiluminescence using ImmunoStar LD reagents (Wako), and images were captured by an LAS-4000 system (FUJIFILM, Tokyo, Japan).

### Transfection and small interfering RNA experiments for FANCJ

The MKN45 cells were cultured in medium without antibiotics for 24 h before transfection at 50–70 % confluence. The cells were transfected with a small interfering RNA (siRNA) oligonucleotide using Lipofectamine RNAiMAX (Invitrogen) in a final siRNA concentration of 40 nmol/l in serum-free Opti-MEM (Invitrogen). After 48 h, the total RNA and proteins were extracted, and the expression levels of the FANCJ mRNA and protein were analyzed by real-time reverse transcription polymerase chain reaction (RT-PCR) and a western blotting analysis, respectively. The siRNA oligonucleotides (Stealth RNAi) and the negative control oligonucleotides (Stealth RNAi siRNA Negative Control) for FANCJ were purchased from Invitrogen.

## Results

### The combination of 5FU and oxaliplatin has synergistic effects against MKN45 cells

**Fig. 1:**
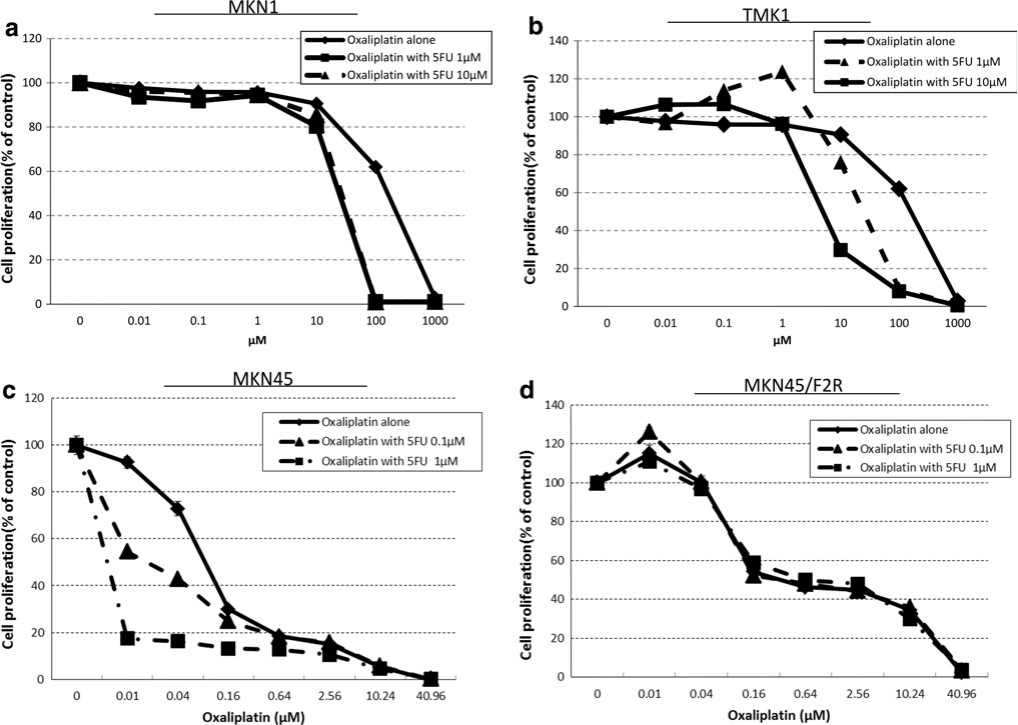
The in vitro sensitivity of the MKN1, TMK1, MKN45 and MKN45/F2R cells to oxaliplatin and/or 5-fluorouracil (*5FU*). **a**, **b**, **d** No synergistic effect was observed at any concentration of 5FU in the MKN1, TMK1, and MKN45/F2R cells. **c** In the MKN45 cells, when 5FU was combined with oxaliplatin, a synergistic effect was observed

To verify that there were synergistic effects of 5FU and oxaliplatin against gastric cancer cells, we performed the MTT assay using 5FU and oxaliplatin in MKN1, TMK1, MKN45, and MKN45/F2R (5FU-resistant) cells (Fig. 1a–d), and calculated the IC50 and the CI using the Loewe additivity model [19] (Table 1). The MKN45/F2R cells were previously established as 5FU-resistant cells in our laboratory [18]. The IC50 of MKN45/F2R cells for 5FU in the present study was 52.4 μM, which is 46.0-fold increased resistance compared with the parent MKN45 cell line, for which the IC50 of 5FU was 1.14 μM, while the major characteristics of these cell lines were consistent, as reported previously [18]. In the MKN45 cells, when 0.1 μM of 5FU was combined with oxaliplatin, the CI was 0.439, which was significantly lower than 1 (*p* < 0.05). This means that the combination had a synergistic effect. Conversely, no synergistic effect was observed in the MKN1, TMK1, and MKN45/F2R cells.

**Table 1:** IC50 values for 5FU and/or oxaliplatin in gastric cancer cells

Drug	MKN1	TMKN1	MKN45	MKN45-F2R
5FU alone	205.50 ± 4.62	297.89 ± 8.92	1.14 ± 0.888	52.4 ± 8.35
Oxaliplatin alone	159.65 ± 4.21	400.66 ± 8.32	0.177 ± 0.00992	2.58 ± 0.311
Oxaliplatin with 0.1 μM 5FU	24.116 ± 0.3425	25.539 ± 1.6378	0.0877 ± 0.00126*	0.317 ± 0.474
Oxaliplatin with 1 μM 5FU	26.315 ± 0.5236	4.99 ± 0.4615	–	0.61 ± 0.526

The 50% inhibitory concentration (IC50) values were calculated from the results of the MTT assay for oxaliplatin and/or 5-fluorouracil (5FU) in the MKN1, TMK1, MKN45, and MKN45/F2R cells. The combination index (CI) was calculated using the Loewe additivity model [19], and a synergistic effect was observed when 0.1 μM of 5FU was combined with oxaliplatin in MKN45 cells (CI = 0.439 ± 0.077**). The IC50 value could not be calculated for these cells when 1 μM of 5FU was combined with oxaliplatin, because the IC50 value was lower than the lowest concentration used in this experiment

* *p* < 0.05 based on Student’s *t*-test

** *p* < 0.05 based on Student’s *t*-test compared to 1

### Changes in ICL repair proteins after 5FU treatment

Oxaliplatin induces its cytotoxic effects primarily by inducing ICLs. We herein examined the differential expression of the proteins involved in ICL repair by a western blotting analysis after treating MKN45 gastric cancer cells with 1 μM, 10 μM, or 100 M of 5FU for 24 h. The proteins examined included FANCJ, BRCA1, MLH1, PMS2, FANCD2, and FANCD1/BRCA2. The FANCJ protein, which is one of the FA gene products, and the tumor suppressor BRCA1 are required to repair DSBs [12, 13]. FANCJ also functions in ICL repair by linking to mismatch repair protein complex MLH1-PMS2 (MutLα) [15]. FANCD1/BRCA2 and FANCD2 are the key proteins in the FA pathway [14]. Interestingly, we observed that the expression of the FANCJ protein was decreased in a dose-dependent manner, and the expression was decreased to 48 % at 100 μM of 5FU compared to the expression level without 5FU. On the other hand, the expression of the BRCA1 protein was increased by 2.1-fold after treatment with 1 μM of 5FU. These changes indicated that FANCJ and BRCA1 functioned to repair the DSBs caused by 5FU, and these proteins were likely to be related to the synergism between 5FU and oxaliplatin, because a deficit of FANCJ protein leads to a failure of ICL repair [15]. None of the expression levels of other proteins involved in DSB or ICL repair, such as MutLα, were changed, or they were only slightly increased after 5FU treatment, and seemed not to be involved in the synergism between 5FU and oxaliplatin.

**Fig. 2:**
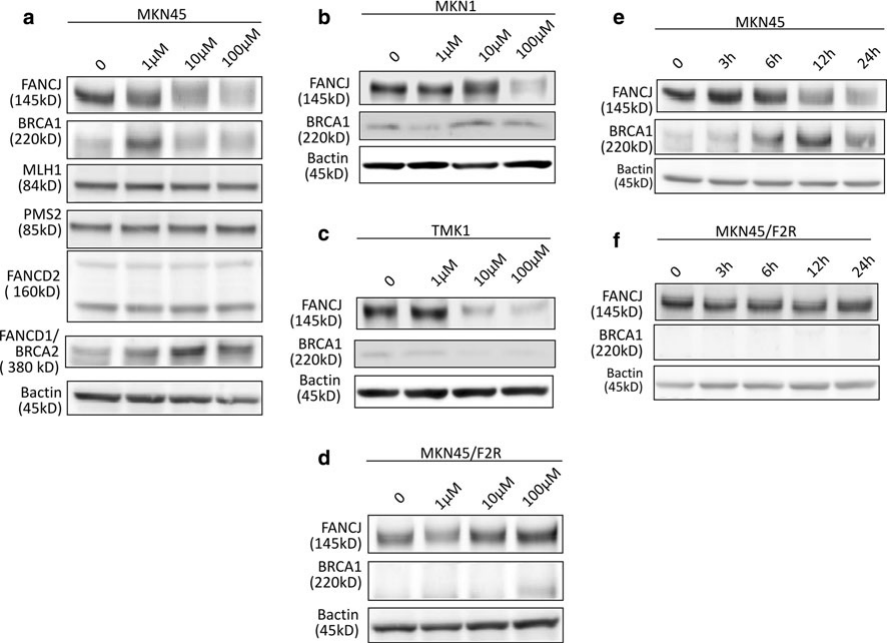
Changes in interstrand crosslink (ICL) repair proteins after 5FU treatment. **a** The results of a western blotting analysis of the expression of FANCJ, BRCA1, MLH1, PMS2, FANCD2, and FANCD1/BRCA2 in MKN45 cells treated with 5FU at 1, 10, and 100 μM for 24 h. **b** The results of the western blotting analysis of FANCJ and BRCA1 in MKN1 cells. **c** The results of the western blotting analysis in TMK1 cells. **d** The results of the western blotting analysis in MKN45/F2R cells. **e** The results of the western blotting analysis of the expression of FANCJ and BRCA1 in MKN45 cells treated with 10 μM of 5FU for 3, 6, 12, and 24 h. **f** The results of the western blotting analysis of the expression of these proteins in MKN45/F2R cells treated with 10 μM of 5FU for 3, 6, 12, and 24 h

We also examined the expressions of FANCJ and BRCA1 in other gastric cancer cell lines, such as MKN1, TMK1, and MKN45/F2R cells. As shown in Fig. 2b–d. The downregulation of FANCJ was reproduced in MKN1 and TMK1 cells, and induction of BRCA1 was also observed in MKN1 cells. In the MKN45/F2R cells, both FANCJ and BRCA1 were unchanged after 5FU treatment.

We then treated MKN45 and MKN45/F2R cells with 10 μM of 5 FU for 3, 6, 12, and 24 h and examined the FANCJ and BRCA1 expression levels by a western blot analysis; as shown in Fig. 2e, f the FANCJ expression in the MKN45 parental cells was decreased and BRCA1 expression was increased in a time-dependent manner. The FANCJ protein was decreased to 48 % of the level of the control after a 24-h treatment, while the expression of BRCA1 was increased by 4.3-fold compared to the control level. These changes were not observed in MKN45/F2R cells.

### DSBs appeared when MKN45 cells were treated with 5FU

It has previously been established that 5FU induces DSBs, and FANCJ functions in DSB repair [12, 13]. Therefore, we examined whether DSBs occurred in MKN45 and MKN45/F2R cells treated with 5FU.

**Fig. 3:**
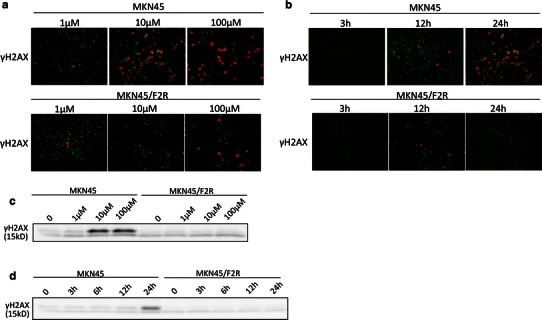
The induction of double-strand breaks (DSBs) in MKN45 and MKN45/F2R cells after treatment with 5FU. An immunofluorescence analysis and a western blotting analysis for phosphor-H2AX, a DSB marker, were performed after treatment with the indicated concentrations of 5FU. **a** The results of the immunofluorescence analysis of MKN45 and MKN45/F2R cells treated with 5FU at concentrations of 1, 10, and 100 μM for 24 h. **b** The results of the immunofluorescence analysis of the MKN45 and MKN45/F2Rcells treated with 10 μM of 5FU for 3 h, 12 h, and 24 h. **c** The results of the western blotting analysis of MKN45 and MKN45/F2R cells treated with 5FU at concentrations of 1, 10, and 100 μM for 24 h. **d** The results of the western blotting analysis of MKN45 and MKN45/F2R cells treated with 10 μM of 5FU for 0, 3, 6,12, and 24 h

To evaluate the DSB status, we performed immunofluorescence studies for γH2AX, which is a marker of DSBs [20, 21]. There were indeed DSBs, which are indicated in red in Fig. 3a. The MKN45 and MKN45/F2R cells were treated with 5FU at concentrations of 1, 10, and 100 μM for 24 h, and we found that DSBs were increased in a dose-dependent manner in the MKN45 parental cells, while this phenomenon was not observed in MKN45/F2R cells (Fig. 3a). We also treated the cells with 10 μM of 5FU for 3, 12, and 24 h, and examined the DSBs (Fig. 3b). As expected, the DSBs were observed in MKN45 parental cells, and they were increased in a time-dependent manner, with DSBs being present in 62 % of the cells after the 24-h treatment. However, no time-dependent DSBs were detected in the MKN45/F2R cells.

Next, we performed a Western blot analysis for γH2AX after 5FU treatment to confirm the increased expression of the protein. The expression of γH2AX was increased by 6.2-fold after treatment with 10 and 100 μM of 5FU for 24 h compared to the control (Fig. 3c), and γH2AX was increased with 10 μM of 5FU in 24-h treatment compared with treatment for other periods (Fig. 3d).

### MLH1 and PMS2 are linked to FANCJ after oxaliplatin treatment

The FANCJ/MutLα interaction is indispensable for ICL repair, and loss of FANCJ leads to failure of ICL repair [15]. To assess the interactions between these proteins and FANCJ after treatment in our cell lines, we performed co-immunoprecipitation studies.

**Fig. 4:**
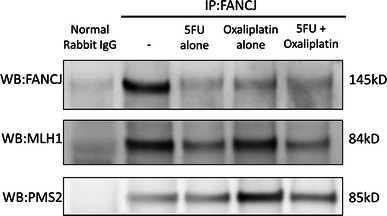
Co-immunoprecipitation (*IP*) with an anti-FANCJ antibody. Co-immunoprecipitation of proteins with FANCJ after treatment of MKN45 cells with 10 μM 5FU and/or 1 μM oxaliplatin for 24 h. After oxaliplatin treatment, both MLH1 and PMS2 were immunoprecipitated to a greater extent than that after 5FU treatment alone, although the amount of FANCJ was decreased. *WB* Western blotting

After MKN45 cells were treated with 10 μM 5FU, 1 μM oxaliplatin,or both agents for 24 h, the cell lysates were immunoprecipitated with an anti-FANCJ antibody, and the presence of co-immunoprecipitated MLH1 and PMS2 was evaluated by a western blot analysis (Fig. 4). After the 5FU treatment, MLH1 and PMS2 were only minimally immunoprecipitated. However, after the oxaliplatin treatment, both MLH1 and PMS2 were immunoprecipitated to a greater extent than after the 5FU treatment, even though the level of FANCJ decreased, suggesting that the amount of MutLα bound to FANCJ was increased after treatment with oxaliplatin in MKN45 cells.

### FANCJ knockdown increases the sensitivity of MKN45 cells to oxaliplatin

**Fig. 5:**
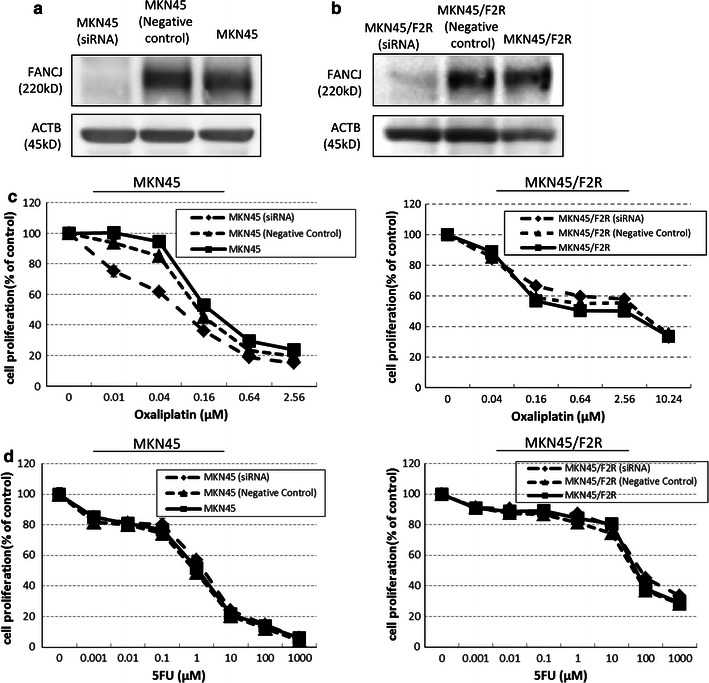
The downregulation of FANCJ after transfection of cells with a small interfering (*si*) RNA oligonucleotide against FANCJ. An siRNA oligonucleotide against FANCJ was transfected into **a** MKN45 and **b** MKN45/F2R cells and the expression of FANCJ was evaluated. The in vitro sensitivity to **c** oxaliplatin or **d** 5FU after siRNA transfection demonstrated that the downregulation of FANCJ increased the sensitivity of MKN45 cells to oxaliplatin

The loss of FANCJ is thought to result in a failure of ICL repair [5], and we found that the FANCJ expression was decreased after 5FU treatment, as described above. Therefore, we hypothesized that the decrease in FANCJ caused by 5FU treatment contributes to the increase in the sensitivity of gastric cancer cells to oxaliplatin. To verify this hypothesis, siRNA directed against FANCJ was transfected into MKN45 and MKN45/F2R cells, and their sensitivity to oxaliplatin and 5FU was analyzed by the MTT assay. Before the sensitivity of the cells was analyzed, the mRNA and protein expression levels of FANCJ were evaluated to confirm that the FANCJ gene was knocked down. As shown in Fig. 5a, in the MKN45 cells transfected with the siRNA oligonucleotide against FANCJ, the expression of FANCJ was decreased to 15.3 % compared to that in the control cells. Similarly, the FANCJ expression in MKN45/FR2 cells was decreased to 25.1 % compared to that in control MKN45/F2R cells (Fig. 5b). Changes in the mRNA expression levels were also confirmed in these cells (data not shown).

We then performed MTT assays for cells treated with oxaliplatin and 5FU. As expected, the IC50 for oxaliplatin in the MKN45 cells after siRNA transfection decreased, to 0.075 μM from 0.177 μM (Fig. 5c; Table 2). On the other hand, the sensitivity of the MKN45 cells to 5FU was not altered (Fig. 5d; Table 2). The sensitivity of MKN45/F2R cells to oxaliplatin and 5FU did not change after siRNA transfection. These results suggest that decreased FANCJ expression increased the sensitivity of MKN45 cells to oxaliplatin, but not to 5FU, while the sensitivity was not altered in 5FU-resistant MKN45/F2R gastric cancer cells.

**Table 2:** IC50 values for oxaliplatin and 5FU in MKN45 and MKN45/F2R cells after siRNA transfection

Cell line (treatment)	IC50 for oxaliplatin (average ± SE)	IC50 for 5FU (average ± SE)
MKN45 (no treatment)	0.177 ± 0.00992	1.14 ± 0.888
MKN45 (negative control)	0.135 ± 0.00175	0.882 ± 0.281
MKN45 (siRNA)	0.075 ± 0.0158*	1.65 ± 0.283
MKN45/F2R (no treatment)	2.58 ± 0.311	52.4 ± 8.35
MKN45/F2R (negative control)	3.75 ± 0.752	44.8 ± 6.02
MKN45/F2R (siRNA)	3.99 ± 0.854	72.0 ± 9.30

MKN45 and MKN45/F2R cells were transfected with a small interfering (si) RNA against FANCJ, and the IC50 values were calculated from the results of the MTT assay for oxaliplatin and/or 5FU. The IC50 for oxaliplatin in the MKN45 cells was significantly decreased after siRNA transfection. On the other hand, the IC50 for 5FU in the MKN45 cells was not altered. The IC50 for oxaliplatin and 5FU in the MKN45/F2R cells did not change after siRNA transfection

* *p* < 0.05 based on Student’s *t*-test, compared with untreated MKN45 or MKN45/F2R cells (no treatment)

## Discussion

Oxaliplatin, a DACH-containing platinum agent, has a spectrum of activity and mechanisms of action and resistance that appear to be different from those of other platinum-containing compounds, notably cisplatin (CDDP) [22]. Moreover, its anticancer effects are optimized when it is administered in combination with other anticancer agents, such as 5-fluorouracil (5FU) [22], S-1 [23, 24], and capecitabine [25, 26] in gastric and colorectal cancers. There have been several reports about the relationship between the FA pathway and oxaliplatin. For example, it was demonstrated that FANCC- and FANCD2-mutant cells were more sensitive to oxaliplatin and CDDP than FANCA-mutant cells, and mono-ubiquitination of FANCD2, which is mediated by the FANCA- and FANCC-containing FA core complex, was not required for platinum resistance [27]. It was also shown that disruptions of FANCC and FANCG caused a 2-fold increase in the sensitivity of RKO cells to oxaliplatin [28].

With regard to the relationship between FANCJ and chemotherapy, Nakanishi et al. reported that there was a correlation between high expression of FANCJ and poor responsiveness of 5FU in colorectal cancer [29]. Our present study is the first to reveal the role of FANCJ in the synergism between 5FU and oxaliplatin. However, other reports about the synergistic effects of oxaliplatin or CDDP in combination with 5FU in vitro also exist. For example, Raymond et al. [10] reported that synergistic antiproliferative effects were observed when oxaliplatin was added to 5FU, and the synergistic effects of these combinations were maintained in the 5FU-resistant colon cancer cell line, HT29-5-FU. Scheithauer and Temsch [30] reported that the addition of CDDP to 5FU/leucovorin (LV) yielded synergistic growth inhibition in some human colon cancer cell lines. Our present study revealed that there were synergistic effects of oxaliplatin in combination with 5FU in the MKN45 gastric cancer cell line, and these effects were also observed with CDDP and 5FU (data not shown).

In our study, γH2AX was increased in MKN45 cells after 5FU treatment. In addition, although BRCA1 protein expression was induced by 5FU treatment, the expression of FANCJ was downregulated. This downregulation may have occurred because the FANCJ protein was bound to newly synthesized BRCA1 to repair the DSBs caused by 5FU treatment, and FANCJ may also have functioned via other mechanisms [31].

In contrast, in the MKN45/F2R 5FU-resistant cells, DSBs did not appear after 5FU treatment, and the expression levels of FANCJ and other proteins were not altered after 5FU treatment. These results confirmed that 5FU downregulated the FANCJ protein in sensitive cells, and this appears to be important for the activity of 5FU. In the present study, γH2AX was not detected after treatment with oxaliplatin to the same extent as it was with 5FU (data not shown), suggesting that the induction of DSBs was a phenomenon specifically related to 5FU treatment.

**Fig. 6:**
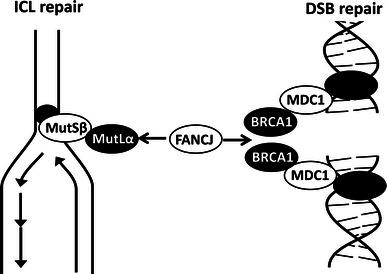
A model of how FANCJ proteins function when cells are treated with 5FU and oxaliplatin. 5FU induces DSBs, while oxaliplatin induces ICLs. Both ICL repair and DSB repair require the FANCJ protein. Because there is a lack of FANCJ when cells are treated with both drugs, there is synergism between 5FU and oxaliplatin

The interaction between FANCJ and MutLα (composed of MLH1 and PMS2) is essential for the ICL response [15]. The ICL is first sensed by MutSβ, but we examined the MutLα (MLH1-PMS2) complex because FANCJ directly binds to MutLα, but not to MutSα or MutSβ, and we considered that the interaction between FANCJ and MutLα was more directly related to the synergism between 5FU and oxaliplatin. As shown in Fig. 2, the expression levels of MLH1 and PMS2 were not altered after 5FU treatment, while there was decreased FANCJ because it was consumed to repair DSBs caused by 5FU treatment. This might have interfered with the repair of ICLs caused by oxaliplatin, thus resulting in the increased sensitivity to oxaliplatin. The involvement of MutSα or β should be examined in the future. A model for the potential involvement of these molecules is illustrated in Fig. 6.

Peng et al. [15] reported that, in the absence of the FANCJ protein, it was impossible to displace MutLα from recombination intermediates, and consequently, the MutLα complex remained stuck to DNA for a longer time period, delaying the exit from the G2/M arrest and enhancing ICL sensitivity [5]. In our study, the level of FANCJ in the MKN45 cells was decreased after 5FU treatment. As would be expected based on the report by Peng et al., the sensitivity of the MKN45 cells to oxaliplatin increased when FANCJ was knocked down by siRNA. We initially tried to force the expression of FANCJ in the cells by transfection, because we wanted to confirm whether the synergism between 5FU and oxaliplatin was reversed by FANCJ overexpression. However, there are various other molecules involved in the synergism, such as BRCA1, MLH1, and so on. This led us to examine the direct effects of FANCJ using an siRNA knockdown system. Our findings suggest that the decrease in FANCJ caused by 5FU treatment leads to an increase in the sensitivity to oxaliplatin, resulting in synergistic cytotoxic effects exerted by the combination of 5FU and oxaliplatin in MKN45 5FU-sensitive cells. In the MKN45/F2R cells, the synergistic effect of oxaliplatin and 5FU was not observed, partly because DSBs did not occur after 5FU treatment in these cells.

In conclusion, the present study provides the first evidence of the role of FANCJ in the synergism between 5FU and oxaliplatin, and can be regarded as providing a rationale for using a combination of fluoropyrimidine and platinum agents for the treatment of gastric carcinomas [22].

## References

[1] Jemal A, Bray F, Center MM, Ferlay J, Ward E, Forman D (2011). Global cancer statistics. CA Cancer J Clin.

[2] Parkin DM, Bray F, Ferlay J, Pisani P (2005). Global cancer statistics, 2002. CA Cancer J Clin.

[3] Koizumi W, Narahara H, Hara T, Takagane A, Akiya T, Takagi M (2008). S-1 plus cisplatin versus S-1 alone for first-line treatment of advanced gastric cancer (SPIRITS trial): a phase III trial. Lancet Oncol.

[4] Colucci G, Gebbia V, Paoletti G, Giuliani F, Caruso M, Gebbia N (2005). Phase III Randomized Trial of FOLFIRI Versus FOLFOX4 in the Treatment of Advanced Colorectal Cancer: a Multicenter Study of the Gruppo Oncologico Dell’Italia Meridionale. J Clin Oncol.

[5] Omura K (2008). Advances in chemotherapy against advanced or metastatic colorectal cancer. Digestion.

[6] Cavanna L, Artioli F, Codignola C (2006). Oxaliplatin in combination with 5-fluorouracil (5-FU) and leucovorin (LV) in patients with metastatic gastric cancer (MGC). Am J Clin Oncol.

[7] Oh SY, Kwon HC, Seo BG, Kim SH, Kim JS, Kim HJ (2007). A phase II study of oxaliplatin with low dose leucovorin and bolus and continuous infusion 5-fluorouracil (modified FOLFOX-4) as first line therapy for patients with advanced gastric cancer. Acta Oncol.

[8] Takashima A, Yamada Y, Nakajima TE, Kato K, Hamaguchi T, Shimada Y (2009). Standard first-line chemotherapy for metastatic gastric cancer in Japan has met the global standard: evidence from recent phase III trials. Gastrointest Cancer Res.

[9] Raymond E, Chaney SG, Taamma A, Cvitkovic E (1998). Oxaliplatin: a review of preclinical and clinical studies. Ann Oncol.

[10] Raymond E, Buquet-Fagot C, Djelloul S, Mester J, Cvitkovic E, Allain P (1997). Antitumor activity of oxaliplatin in combination with 5-fluorouracil and the thymidylate synthase inhibitor AG337 in human colon, breast, and ovarian cancers. Anticancer Drugs.

[11] Hiom K (2010). FANCJ: solving problems in DNA replication. DNA Repair.

[12] Wu Y, Brosh RM (2009). FANCJ helicase operates in the Fanconi anemia DNA repair pathway and the response to replicational stress. Curr Mol Med.

[13] Litman R, Peng M, Jin Z, Zhang F, Zhang J, Powell S (2005). BACH1 is critical for homologous recombination and appears to be the Fanconi anemia gene product FANCJ. Cancer Cell.

[14] Moldovan GL, D’Andrea AD (2009). How the Fanconi anemia pathway guards the genome. Annu Rev Genet.

[15] Peng M, Litman R, Xie J, Sharma S, Brosh RM, Cantor SB (2007). The FANCJ/MutLa interaction is required for correction of the cross-link response in FA-J cells. EMBO J.

[16] Matuo R, Sousa FG, Escargueil AE, Soares DG, Grivicich I, Saffi J (2010). DNA repair pathways involved in repair of lesions induced by 5-fluorouracil and its active metabolite FdUMP. Biochem Pharmacol.

[17] van der Wilt CL, Kuiper CM, Peters GJ (1999). Combination studies of antifolates with 5-fluorouracil in colon cancer cell lines. Oncol Res.

[18] Tsutani Y, Yoshida K, Sanada Y, Wada Y, Konishi K, Fukushima M (2008). Decreased orotate phosphoribosyltransferase activity produces 5-fluorouracil resistance in a human gastric cancer cell line. Oncol Rep.

[19] Tallarida RJ (2001). Drug synergism: its detection and applications. J Pharmacol Exp Ther.

[20] Rogakou EP, Pilch DR, Orr AH, Ivanova VS, Bonner WM (1998). DNA double-stranded breaks induce histone H2AX phosphorylation on serine 139. J Biol Chem.

[21] Mah LJ, El-Osta A, Karagiannis TC (2010). GammaH2AX: a sensitive molecular marker of DNA damage and repair. Leukemia.

[22] Raymond E, Faivre S, Chaney S, Woynarowski J, Cvitkovic E (2002). Cellular and molecular pharmacology of oxaliplatin. Mol Cancer Ther.

[23] Koizumi W, Takiuchi H, Yamada Y, Boku N, Fuse N, Muro K (2010). Phase II study of oxaliplatin plus S-1 as first-line treatment for advanced gastric cancer (G-SOX study). Ann Oncol.

[24] Oh SY, Kwon HC, Jeong SH, Joo YT, Lee YJ, Hee Cho S, et al. A phase II study of S-1 and oxaliplatin (SOx) combination chemotherapy as a first-line therapy for patients with advanced gastric cancer. Invest New Drugs. 2010. doi:10.1007/s10637-010-9507-2. 10.1007/s10637-010-9507-2 20706861

[25] Rothenberg ML, Cox JV, Butts C, Navarro M, Bang YJ, Goel R (2008). Capecitabine plus oxaliplatin (XELOX) versus 5-fluorouracil/folinic acid plus oxaliplatin (FOLFOX-4) as second-line therapy in metastatic colorectal cancer: a randomized phase III noninferiority study. Ann Oncol.

[26] Ducreux M, Bennouna J, Hebbar M, Ychou M, Lledo G, Conroy T (2011). Capecitabine plus oxaliplatin (XELOX) versus 5-fluorouracil/leucovorin plus oxaliplatin (FOLFOX-6) as first-line treatment for metastatic colorectal cancer. Int J Cancer.

[27] Kachnic LA, Li L, Fournier L, Willers H (2010). Fanconi anemia pathway heterogeneity revealed by cisplatin and oxaliplatin treatments. Cancer Lett.

[28] Gallmeier E, Calhoun ES, Rago C, Brody JR, Cunningham SC, Hucl T (2006). Targeted disruption of FANCC and FANCG in human cancer provides a preclinical model for specific therapeutic options. Gastroenterology.

[29] Nakanishi R, Kitao H, Fujinaka Y, Yamashita N, Iimori M, Tokunaga E, et al. FANCJ expression predicts the response to 5-fluorouracil-based chemotherapy in MLH1-proficient colorectal cancer. Ann Surg Oncol. 2012 (epub ahead of print). 10.1245/s10434-012-2349-8 22526901

[30] Scheithauer W, Temsch EM (1989). A study of various strategies to enhance the cytotoxic activity of 5-fluorouracil/leucovorin in human colorectal cancer cell lines. Anticancer Res.

[31] Xie J, Litman R, Wang S, Peng M, Guillemette S, Rooney T (2010). Targeting the FANCJ-BRCA1 interaction promotes a switch from recombination to poleta-dependent bypass. Oncogene.

